# Loneliness and Mental Health: The Mediating Effect of Perceived Social Support

**DOI:** 10.3390/ijerph182211963

**Published:** 2021-11-14

**Authors:** Elody Hutten, Ellen M. M. Jongen, Anique E. C. C. Vos, Anja J. H. C. van den Hout, Jacques J. D. M. van Lankveld

**Affiliations:** 1 Department of Clinical Psychology, Open Universiteit, 6401 DL Heerlen, The Netherlands; ellen.jongen@ou.nl (E.M.M.J.); Jacques.vanLankveld@ou.nl (J.J.D.M.v.L.); 2 Department of Clinical and Medical Psychology, Zuyderland Medical Center, 6419 PC Heerlen, The Netherlands; an.vos@zuyderland.nl (A.E.C.C.V.); a.vandenhout@zuyderland.nl (A.J.H.C.v.d.H.)

**Keywords:** loneliness, social support, anxiety, depression, somatic symptoms, somatic symptom disorder

## Abstract

Social connectedness is a fundamental human need. The Evolutionary Theory of Loneliness (ETL) predicts that a lack of social connectedness has long-term mental and physical health consequences. Social support is a potential mechanism through which loneliness influences health. The present cross-sectional study examined the relationship between loneliness and mental health, and the mediating effects of social support in a Dutch adult sample (N = 187, age 20 to 70). The health variables included in the study are anxiety, depression, somatic symptoms as measured by the SCL-90, and the DSM-5 diagnosis somatic symptom disorder. The results indicated that social support partially mediated the relationship between loneliness and anxiety, depression, and somatic symptoms. These results indicate that social support partially explains the relationship between loneliness and physical and mental health issues. The relationship between loneliness and being diagnosed with somatic symptom disorder was not mediated by social support. This suggests that the mechanisms through which loneliness relates to either somatic symptoms or somatic symptom disorder are different.

## 1. Introduction

Social connectedness is a fundamental human need [1]. When this need to belong is not met, feelings of loneliness can occur. Loneliness is the distressing feeling associated with a discrepancy between a person’s desired and actual levels of social relations [2]. Therefore, loneliness is the subjective experience of a quantitative or qualitative deficiency in one’s social relationships. Previous studies have revealed that loneliness is relatively common, with between 10.5% to 43.5% of the general population in developed countries reporting some degree of loneliness [3,4,5,6,7]. Furthermore, loneliness has been associated with negative outcomes such as increased mortality risk [8]. Together, the high prevalence rates and negative consequences of loneliness stress the importance of research on loneliness and its health consequences.

The Evolutionary Theory of Loneliness (ETL) predicts that loneliness has long-term mental and physical health consequences [9]. According to this theory, perceived social isolation (i.e., loneliness) has a signaling function, similar to physical pain [10,11]. Social connections have been deemed to be the primary behavioral adaptation of human and nonhuman primates and provide protection from the threat of predation and scarcity of resources [12]. If an individual becomes socially isolated, he/she becomes deprived of the protection that sociality brings. To promote self-preservation, loneliness causes hypervigilance towards social threat [13,14,15]. This attention bias towards social threat can help sustain loneliness by causing lonely individuals to have more negative social expectations leading to self-defeating social behavior [11]. This self-reinforcing loneliness loop has been argued to cause increased physical and mental health risk in the long-term [9,11].

Consistent with the ETL, loneliness has been associated with poorer mental and physical health [16,17,18,19]. Among the mental health issues associated with loneliness are anxiety and depression [16,17,18,19,20]. Anxiety is a long-term trait marked by a non-adaptive hypervigilance and overestimation of the potential for threat [21]. Previous studies have established a relationship between loneliness and anxiety symptoms and disorders among adolescents [22,23], students [14], adults [18], and older adults [24]. Depression is characterized by a loss of positive affect that manifests itself in withdrawal, avoidance, and loss of activity [25]. Symptoms associated with depression include lack of interest and pleasure from activity, sleep disturbance, and feelings of worthlessness [26]. The relationship between loneliness and depression is well documented in the literature and has been studied in adolescent [22,23,27,28], adult [18], and older adult [24] samples. Furthermore, loneliness is associated with somatic symptoms [3,29]. Previous studies have established a relationship between loneliness and somatic symptoms among adults [3] and older adults [29]. Somatic symptoms are physical in nature and are perceived as worrisome or unpleasant [30]. Somatic symptoms include headaches, pain, and dizziness. When somatic symptoms are persistent, clinically significant, and influence the individual’s thoughts, feelings, and behavior, they can develop into a mental disorder [31].

Somatic symptom disorder (SSD) is a mental disorder recognized by the DSM-5 [26]. SSD replaces previously recognized somatoform disorders including somatization disorder, undifferentiated somatoform disorder, and hypochondriasis. The diagnostic criteria for SSD are the presence of (1) one or more somatic symptoms that are distressing or disturb daily life; (2) one or more excessive thoughts, feelings, or behaviors related to the somatic symptoms that manifest themselves for example in disproportionate catastrophizing and high levels of anxiety; and (3) the persistent experience of somatic symptoms that lasts for at least 6 months. Although the relationship between SSD and loneliness has not been investigated to date, previous studies have established that somatoform disorders, such as irritable bowel syndrome, fibromyalgia, and undifferentiated somatoform disorder, are associated with higher levels of loneliness, and feelings of social rejection and invalidation [32,33,34]. Although previous studies have established a relationship between loneliness and mental and physical health, less is known about the mechanisms through which loneliness is related to health.

Loneliness has been argued to generally cause physical and mental health issues indirectly through mediating variables [35,36]. Previous research has been able to identify mediating variables that transmit the relationship between loneliness and physical [37] and mental health [35,37,38,39,40]. An example of a mediator identified in the literature is social support. Social support is conceptually close, but distinct from loneliness. Social support refers to the degree to which an individual receives help, guidance, comfort, and information from one’s social relations [41]. Social support can occur in different domains including informational, instrumental, and emotional support [42,43]. Furthermore, the literature on social support generally distinguishes received and perceived social support. Whereas received social support refers to the quantity of support one receives [44], perceived social support refers to the adequacy and availability of the social support one receives [45]. Previous studies have revealed that the relationship between perceived social support and mental and physical health is stronger than the relationship between received social support and mental and physical health [46,47,48]. 

Cohen and Wills [49] argued that social support might contribute to physical and mental health because social resources are a protective factor for health issues (i.e., the main-effect model) or a buffer against stressful events (i.e., the buffering model). Consistent with these models, social support has been associated with physical [50] and mental [19] health. A systematic review by Wang et al. [19] revealed that lower levels of perceived social support were associated with worse depressive symptoms and lower recovery rates. Furthermore, Wang et al. [19] report preliminary evidence that links lower perceived social support to anxiety symptoms. Although perceived social support has also been associated with lower somatic symptoms among older adults [51] and patients with somatic symptom disorder [52], another study has found no relationship between both constructs in a sample of ethnic minority military recruits [53].

Although previous studies have demonstrated that social support is a mediating variable that (partially) explains how loneliness may lead to physical and mental health problems [35,38], these studies have two important limitations. First, both studies have only focused on the mediating role of social support in the relationship between loneliness and depression. Therefore, whether social support serves as a mediating mechanism in the relationship between loneliness and mental health issues other than depression is lacking. Furthermore, these studies have been conducted among older adults in China [38] and Malaysia [35]. Thus, the generalizability of these results is limited. The present study aims to address these issues by investigating whether perceived social support acts as a mediating mechanism in the relationship between loneliness and mental health issues (i.e., anxiety, depression, somatic symptoms, and somatic symptom disorder) in a Dutch adult sample. Thus, the present study contributes to the literature on the mental health consequences of loneliness by providing an explanation as to how and why loneliness may lead to physical and mental health issues. Please note that the mediating role of social support in the relationship between loneliness and mental health were investigated using a cross-sectional design. Therefore, conclusions on mediation have to be carefully drawn.

## 2. Materials and Methods

### 2.1. Sample

The data used in the present study were obtained from a larger cross-sectional study on somatic symptom disorder conducted at the department of Medical Psychology of the Zuyderland Medical Center located in Heerlen, the Netherlands. The sample of this study includes both a patient group which consists of individuals diagnosed with DSM-5 somatic symptom disorder and a control group which consists of respondents without a mental disorder. Recruitment of patients occurred among individuals with medically unexplained symptoms who were referred to the department of Medical Psychology of the Zuyderland Medical Center. Individuals who agreed to participate in the study were asked to complete the De Jong Gierveld Loneliness scale and the Social Support List during the psychodiagnostic process in addition to the regular instruments (including the Symptom Checklist-90). Control group participants were recruited among the researchers’ social network. The final sample size used in the study was N = 187 (63.1% female, age range from 20 to 70 with Mage = 43.0 and SDage = 13.4) consisting of 75 patients diagnosed with somatic symptom disorder and 112 healthy controls.

### 2.2. Measures

Anxiety, depression, and somatization were assessed using the corresponding subscales of the Dutch version of the Symptom Checklist-90 (SCL-90) [54]. Anxiety was assessed using a subscale containing 10 symptoms for anxiety (e.g., “worrying too much about things”; α = 0.93). The subscale for depression consists of 16 symptoms related to depression (e.g., “feeling low in energy or slowed down”; α = 0.96). The subscale for somatization consists of 12 physical symptoms (e.g., “dizziness”; α = 0.93). Participants rated whether they experienced any of these symptoms on a 5-point scale ranging from ‘not at all’ (1) to ‘extremely’ (5). Higher scores indicate a higher symptom level. Somatic symptom disorder was included as a dichotomous variable with 0 = no DSM-5 diagnosis of somatic symptom disorder and 1 = DSM-5 diagnosis of somatic symptom disorder. 

Perceived social support was assessed using the ‘discrepancy’ measure of the Social Support List (SSL) [55,56]. The SSL consists of 34 items (e.g., measuring both the frequency of social support (SSL-I) and the discrepancy between the desired and received level of social support (SSL-D). The SSL measures six domains of social support: everyday emotional support, emotional support in case of problems, appreciation support, instrumental support, social companionship, and informational support. Participants rated the items of the SSL-D on a 4-point scale ranging from ‘I miss it; would like more’ (1) to ‘happens too often; wish it was less’ (4; α = 0.95). Scores of 3 and 4 were recoded to 1 and scores of 1 were recoded to 3 to ensure that the score reflects an experienced shortage in social support. Therefore, a higher score indicates a perceived deficit in social support.

Loneliness was measured using the De Jong Gierveld Loneliness scale (De Jong Gierveld Eenzaamheidsschaal) [57]. The scale consists of 11 items assessing a discrepancy between a person’s actual and desired level of social relations (e.g., ‘I miss having a really close friend’; α = 0.90). Participants rated the items on a 5-point scale ranging from ‘no!’ (1) to ‘yes!’ (5).

The present study included five demographic variables as covariates: sex, age, educational attainment, employment status, and marital status. Sex was included as a dummy variable with 0 = female and 1 = male and age was included as a continuous variable. Educational attainment was recoded into a dummy variable with 0 = not higher educated and 1 = higher educated. Having a paid job was recoded into a dummy variable with 0 = not having a paid job and 1 = having a paid job. Finally, marital status was recoded into a dummy variable with 0 = not single and 1 = single.

### 2.3. Data Analysis

Averaged scores were included in the data analysis. Descriptive statistics and Pearson correlations were calculated for the variables included in the present study. The mediating role of perceived social support in the relationship between loneliness and mental health was investigated using model 4 of the PROCESS macro v.3.4.1 [58]. This macro is based on the bootstrap method (n = 5000) which has been argued to yield the most accurate results for indirect effects [59]. More specifically, four regression analyses were performed with dependent variables of anxiety, depression, somatization, and somatic symptom disorder. All regression analyses included loneliness as independent variable and perceived social support as mediator. 

Previous research has revealed that sex [60,61], age [62,63,64], educational attainment [65,66], work [67,68,69,70,71,72], and marital status [73] are associated with mental health. Therefore, these factors were included as covariates in the regression analyses. All analyses were performed using IBM SPSS Statistics 25.

## 3. Results

**Figure 1:**
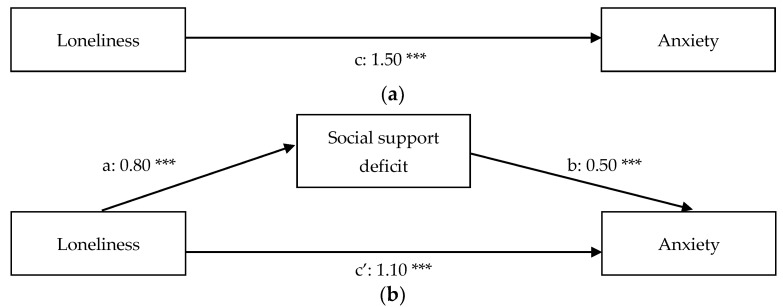
(**a**) Total relationship between loneliness and anxiety. (**b**) Mediation of the relationship between loneliness and anxiety. *** *p* < 0.01.

**Figure 2:**
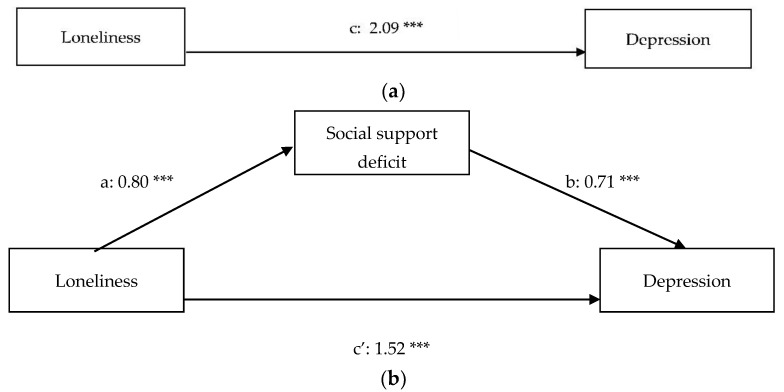
(**a**) Total relationship between loneliness and depression. (**b**) Mediation of the relationship between loneliness and depression. *** *p* < 0.01.

**Figure 3:**
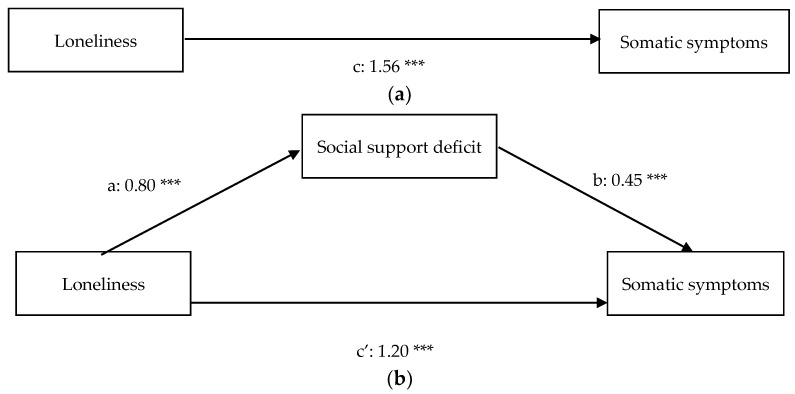
(**a**) Total relationship between loneliness and somatic symptoms. (**b**) Mediation of the relationship between loneliness and somatic symptoms. *** *p* < 0.01.

**Figure 4:**
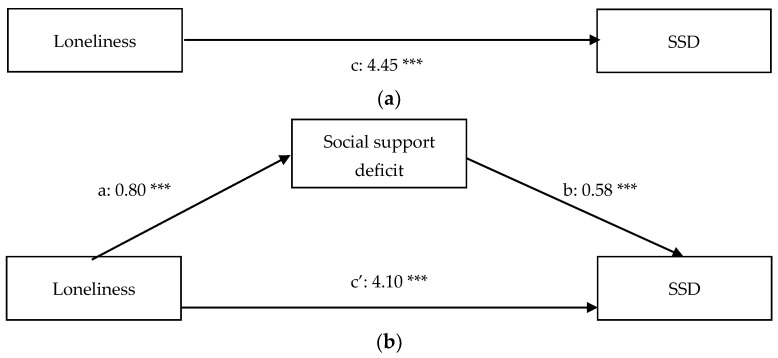
(**a**) Total relationship between loneliness and somatic symptom disorder (SSD). (**b**) Mediation of the relationship between loneliness and somatic symptom disorder (SSD). *** *p* < 0.01.

Table 1 contains the descriptive statistics and Pearson correlations of the variables included in the present study. The results of the regression analyses can be found in Table 2 (before inclusion of the covariates) and Table 3 (after inclusion of the covariates) and path diagrams of the regression models (after inclusion of the covariates) can be found in Figure 1a,b (anxiety), Figure 2a,b (depression), Figure 3a,b (somatic symptoms), and Figure 4a,b (somatic symptom disorder). The R^2^ of the four regression models after inclusion of the covariates are 0.53 for the model predicting anxiety, 0.63 for the model predicting depression, 0.55 for the model predicting somatic symptoms, and 0.50 for the model predicting somatic symptom disorder. Therefore, the effect sizes of our regression models are substantial [74].

The results of the regression analysis with anxiety as the dependent variable shows that loneliness is a predictor of anxiety (path c: β = 1.50, *p* < 0.01). The mediation analysis reveals that the relation between loneliness and anxiety is partially mediated by social support. There is a positive, direct effect of loneliness on anxiety (path c’: β = 1.10, *p* < 0.01) and a positive, indirect effect of loneliness on anxiety through perceived deficiency in social support (β = 0.40, *p* < 0.01). Higher educational attainment (β = −0.23; t (179) = −2.43; *p* < 0.05) and being employed (β = −0.34; t (179) = −3.28; *p* < 0.01) were negatively associated with anxiety.

The results of the regression analysis with depression as the dependent variable shows that loneliness is a predictor of depression (path c: β = 2.09, *p* < 0.01). The mediation analysis reveals that the relation between loneliness and depression is partially mediated by social support. There is a positive, direct effect of loneliness on depression (path c’: β = 1.52, *p* < 0.01) and a positive, indirect effect of loneliness on depression through perceived deficiency in social support (β = 0.57, *p* < 0.01). Being employed (β = −0.43; t (179) = −3.99; *p* < 0.01) was negatively associated with depression.

The results of the regression analysis with somatic symptoms as the dependent variable shows that loneliness is a predictor of somatization (path c: β = 1.56, *p* < 0.01). The mediation analysis reveals that the relation between loneliness and somatic symptoms is partially mediated by social support. There is a positive, direct effect of loneliness on somatization (path c’: β = 1.20, *p* < 0.01) and a positive, indirect effect of loneliness on somatization through perceived deficiency in social support (β = 0.36, *p* < 0.01). Higher educational attainment (β = −0.38; t (179) = −3.63; *p* < 0.01) and being employed (β = −0.43; t (179) = −3.88; *p* < 0.01) were negatively associated with somatization. 

The results of the logistic regression analysis with DSM-5 diagnosis of somatic symptom disorder as the independent variable shows that loneliness is a predictor of DSM-5 diagnosis of somatic symptom disorder (path c: β = 4.45, *p* < 0.01). The mediation analysis reveals that the relation between loneliness and a DSM-5 diagnosis of somatic symptom disorder is not partially mediated by the social support. Higher educational attainment (β = −2.15; z (179) = −4.44; *p* < 0.00) and being employed (β = −2.34; z (179) = −4.53; *p* < 0.00) were negatively associated with somatic symptom disorder.

## 4. Discussion

The Evolutionary Theory of Loneliness (ETL) predicts that loneliness has long-term health consequences [9]. In addition, mechanisms have been suggested that might explain the relationship between loneliness and health issues [35,36]. Although previous studies have established that social support might partially explain the relationship between loneliness and mental health [35,38], these studies have two limitations: First, these studies have only investigated the relationship between loneliness and depression. Second, the generalizability of these studies is limited because they have been conducted in older adult, Asian samples. The objective of the present study was to investigate whether perceived social support can explain relationships between loneliness on the one hand and either mental health (i.e., anxiety and depression), physical (i.e., somatic symptoms) health, or somatic symptom disorder on the other hand.

The present study found a relationship between loneliness and mental health. More specifically, higher levels of loneliness were associated with more anxiety, depressive symptoms, and somatic symptoms. Furthermore, the results of the present study reveal that loneliness is associated with a higher likelihood of being diagnosed with somatic symptom disorder. These results are in line with previous research that established a positive relationship between loneliness and depression and anxiety [18,24], somatic symptoms [3,29], and somatoform disorders [32,33,34]. The ETL [9] provides a potential explanation for the positive associations between loneliness and mental health observed in the present study. According to this theory, loneliness triggers an attention bias towards social threat that causes a self-defeating loop of loneliness. This loneliness loop is associated with negative affect such as feelings of fear and sadness [13,14,75]. Persistent negative affect may evolve into mental health issues [76]. Furthermore, the self-reinforcing loneliness loop triggers neurobiological and behavioral changes that deteriorate health [76]. These health-related changes include diminished health behaviors, sleep, and physiological and immunological functioning. These effects are hypothesized to reduce the lonely individual’s resilience and might make them more vulnerable to the development of somatic symptoms. Although the ETL predicts that loneliness causes mental health issues, previous studies have established that the relationship between loneliness and mental health is bi-directional [18,24]. Given the cross-sectional design of the present study, we are unable to shed further light on the direction of the relationship between loneliness and mental health.

Educational attainment and employment status were found to be protective factors for mental health issues. The social determinants of health are well established [69]. This body of literature has revealed that having a higher socioeconomic position [65,66,69] and work [67,68,69,70,71,72] are generally beneficial to health and well-being. In the present study, higher educational attainment was associated with lower levels of anxiety and somatic symptoms and a lower likelihood of being diagnosed with somatic symptom disorder consistent with the existing body of literature. Several explanations have been proposed for this relationship between education and health. Lower levels of education might cause health issues through increased stress levels associated lower socioeconomic status [77,78], through less effective coping strategies and health behavior [65] or cognitive and information differences associated with different education levels [78]. However, a reverse causal relationship has been proposed as well [65]. In the present study, being employed was associated with lower levels of anxiety, depression, and somatic symptoms and a lower likelihood of being diagnosed with somatic symptom disorder in line with the existing body of literature. The health benefits of employment have been explained by economic resources, social factors such as interactions with others and social status, and purpose and achievement [70,79].

While the cross-sectional design of the present study does not allow causal inferences, the results of the mediation analyses suggest that the relationship between loneliness and mental health (i.e., anxiety, depression, and somatic symptoms) might be partially mediated by a perceived deficiency in social support. This result is in line with previous studies that suggested a mediating role of social support among older adults [35,38]. Lonely individuals perceive their social environment differently than non-lonely individuals [80]. For example, loneliness is associated with more negative perceptions of others [81,82]. Consequently, lonely individuals’ negatively skewed perceptions of their social environment might cause them to perceive the social support they receive as more deficient as well. Consequently, these perceived deficits in received social support contribute to physical and mental health issues. The positive relationship observed between a perceived deficiency in social support and mental health is in line with previous research [48,83]. Consistent with the main-effect hypothesis of social support, these results suggest that social support has a beneficial effect on health independent of stressors [49]. This protective effect of social support stems from the fact that it provides individuals with socially rewarding roles that support positive affect and, consequently, promote mental health. Furthermore, social support might promote physical health through emotionally-induced improvements in immune system functioning. 

Although the regression analyses suggest that the relationship between loneliness and anxiety, depression, and somatic symptoms might be partially mediated by a perceived deficiency in social support, no such results were found for the association between loneliness and somatic symptom disorder. These results suggest that the relationship between loneliness and somatic symptom disorder might be different from the relationship between loneliness and anxiety, depression, and somatic symptoms. Somatic symptom disorder is not only characterized by unpleasant physical symptoms (e.g., pain and dizziness) but by excessive or abnormal thoughts, feelings, and behavior as well [26]. The complexity resulting from the combination of physical and psychological symptoms could be an explanation for the different results obtained for the relationships between loneliness and somatic symptoms on the one hand and loneliness and SSD on the other hand. Adding to the complexity of somatic symptom disorder are its different manifestations due to the fact that it is the consolidation of several recognized disorders (e.g., somatization disorder, undifferentiated somatoform disorder, and hypochondriasis) and can be associated with one or multiple persistent bodily symptoms.

The present study is one of the first studies to investigate the mediating role of social support in the relationship between loneliness and a broad range of mental health consequences (i.e., anxiety, depression, somatic symptoms, and somatic symptom disorder). However, two limitations should be kept in mind when interpreting the results: First, the generalizability of the results might be limited due to the fact that we used non-random sampling methods. Second, an important limitation of the present study is the fact that cross-sectional data were used. Therefore, no causal inferences can be drawn. Future research could investigate the mediating role of social support in the relationship between loneliness and mental health by using longitudinal or experimental data. Such research is warranted because evidence for a causal relationship between loneliness and mental health through social support could have practical implications for interventions that aim to prevent or reduce mental health issues in lonely individuals. Early interventions that effectively reduce feelings of loneliness [84,85] might prevent lonely individuals from developing health issues. Aside from targeting loneliness, a mediation effect of social support on loneliness and mental health would suggest that such interventions could target social support as well [86,87].

The results of the present study reveal that the associations between loneliness and mental health remain significant after taking social support into account. The observed associations between loneliness and mental health suggest that interventions that effectively reduce loneliness might also improve health. However, research addressing the health effects of loneliness interventions is scarce and more research is needed to understand how and whether such interventions enhance health [88]. Furthermore, the association between loneliness and mental health could indicate the existence of other mediator variables. The literature on loneliness has suggested other mechanisms through which loneliness causes physical and mental health issues, such as sleep disturbance, physiological functioning, stress responses, immune functioning, and health behavior [36,88].

Finally, the generalizability of the results might be limited because we used non-random sampling methods.

## 5. Conclusions

Although previous studies have consistently reported associations between loneliness and mental and physical health, less is known about the mechanisms through which loneliness is related to health. The mediation analyses of the present study suggest that perceived social support might partially explain the relationship between loneliness and anxiety, depression, and somatic symptoms. This implies that interventions that effectively make up an individual’s perceived deficiency in social support might prevent or reduce mental and physical health issues in the lonely. Given the limitations of the current study’s design, future research using longitudinal or experimental data is however necessary to bolster the conclusion that social support acts as mechanism in the relationship between loneliness and mental health. Furthermore, the results suggest that the relation between loneliness and a DSM-5 diagnosis of somatic symptom disorder is not partially explained by social support.

## Figures and Tables

**Table 1:** Descriptives and correlations of the measures included in the analyses.

	M(SD) or %	Min	Max	Kurtosis	Skewness	1.	2.	3.	4.	5.	6.	7.	8.	9.	10.	11.
1. Loneliness	0.25 (0.30)	0.00	1.00	0.15	1.13	1	0.70 **	0.67 **	0.74 **	0.66 **	0.58 **	0.03	0.14	−0.32 **	−0.38 **	−0.30 **
2. Social support deficit	1.34 (0.37)	1.00	2.68	1.16	1.32		1	0.59 **	0.66 **	0.57 **	0.44 **	0.07	0.11	−0.27 **	−0.30 **	0.28 **
3. Anxiety	1.72 (0.84)	1.00	4.90	1.58	1.40			1	0.89 **	0.87 **	0.73 **	−0.01	0.12	−0.38 **	−0.44 **	0.22 **
4. Depression	1.91 (0.99)	1.00	4.63	0.10	1.11				1	0.86 **	0.74 **	0.00	0.12	−0.35 **	−0.46 **	0.21 **
5. Somatic symptoms	1.91 (0.94)	1.00	4.75	−0.36	0.92					1	0.83 **	−0.03	0.15 *	−0.44 **	−0.47 **	0.21 **
6. SSD	40.1%										1	0.01	0.13	−0.51 **	−0.54 **	0.20 **
7. Sex	36.9%											1	0.08	0.07	0.04	−0.05
8. Age	43.99 (13.42)												1	−0.08	−0.01	−0.20 **
9. Education	53.5%													1	0.28 **	−0.02
10. Having a paid job	66.8%														1	−0.27 **
11. Being single	27.8%															1

Note: ** *p* < 0.01, * *p* < 0.05.

**Table 2:** Direct and indirect effects and 95% confidence intervals for the mediation analyses before inclusion of the covariates.

	Anxiety			Depression			Somatic Symptoms			Somatic Symptom Disorder		
	Estimated Effect	95% CI		Estimated Effect	95% CI		Estimated Effect	95% CI		Estimated Effect	95% CI	
		Lower Bounds	Upper Bounds		Lower Bounds	Upper Bounds		Lower Bounds	Upper Bounds		Lower Bounds	Upper Bounds
Direct effect												
Loneliness → social support deficit	0.87 **	0.74	0.99	0.87 **	0.74	0.99	0.87 **	0.74	0.99	0.87 **	0.74	0.99
Loneliness → outcome	1.37 **	0.96	1.78	1.76 **	1.34	2.19	1.60 **	1.13	2.06	4.74 **	2.92	6.57
Social support deficit → outcome	0.55 **	0.21	0.88	0.74 **	0.39	1.08	0.52 **	0.10	0.81	0.77	−0.60	2.15
Indirect effect												
Loneliness → social support deficit → outcome	0.47 **	0.12	0.83	0.64 **	0.22	1.09	0.45 **	0.05	0.86	0.67	−0.69	1.97
*N*			187			187			187			187
R^2^			0.47			0.58			0.46			0.28

Note: ** *p* < 0.01, * *p* < 0.05.

**Table 3:** Direct and indirect effects and 95% confidence intervals for the mediation analyses after inclusion of the covariates.

	Anxiety			Depression			Somatic Symptoms			Somatic Symptom Disorder		
	Estimated Effect	95% CI		Estimated Effect	95% CI		Estimated Effect	95% CI		Estimated Effect	95% CI	
		Lower Bounds	Upper Bounds		Lower Bounds	Upper Bounds		Lower Bounds	Upper Bounds		Lower Bounds	Upper Bounds
Direct effect												
Loneliness → social support deficit	0.80 **	0.65	0.95	0.80 **	0.65	0.95	0.80 **	0.65	0.95	0.80 **	0.65	0.95
Loneliness → outcome	1.10 **	0.68	1.53	1.52 **	1.08	1.95	1.20 **	0.74	1.65	4.10 **	1.94	6.25
Social support deficit → outcome	0.50 **	0.18	0.83	0.71 **	0.38	1.05	0.45 *	0.10	0.81	0.58	−1.11	2.27
Indirect effect												
Loneliness → social support deficit → outcome	0.40 **	0.08	0.74	0.57 **	0.21	0.99	0.36 **	0.01	0.72	0.46	−1.31	2.36
*N*			187			187			187			187
R^2^			0.53			0.63			0.55			0.50

Note: ** *p* < 0.01, * *p* < 0.05.

## Data Availability

The datasets presented in this article are not readily available because the data are part of a larger cross-sectional study on somatic symptom disorder conducted at the department of Medical Psychology of the Zuyderland Medical Center located in Heerlen, the Netherlands. Requests to access the datasets should be directed to contacts at: an.vos@zuyderland.nl.
